# Optimization of Antioxidant Activity of Compounds Generated during Ginseng Extract Fermentation Supplemented with *Lactobacillus*

**DOI:** 10.3390/molecules29061265

**Published:** 2024-03-13

**Authors:** Shuiqing Lin, Yuxiao Wu, Qian Huang, Zhiting Liu, Juan Xu, Ruifeng Ji, Natalia V. Slovesnova, Xin He, Lin Zhou

**Affiliations:** 1 School of Traditional Chinese Medicine, Guangdong Pharmaceutical University, Guangzhou 510006, China; 2112248160@gdpu.edu.cn (S.L.); liuzhiting@gdpu.edu.cn (Z.L.); jiruifeng0708@163.com (R.J.); 2 Guangdong Provincial Key Laboratory of Advanced Drug Delivery, Guangdong Provincial Engineering Center of Topical Precise Drug Delivery System, School of Life Sciences and Biopharmaceutics, Guangdong Pharmaceutical University, Guangzhou 510006, China; 2112140186@gdpu.edu.cn (Y.W.); 1112342004@gdpu.edu.cn (Q.H.); 3 Lafang China Co., Ltd., Shantou 515000, China; xj@laf.cn; 4 Department of Pharmacy, Ural State Medical University, Repina st.3, 620028 Yekaterinburg, Russia; saarge@mail.ru

**Keywords:** ginseng stem and leaf, *Lactobacillus*, antioxidant activity, fermentation, cell viability

## Abstract

Ginseng holds high medicinal and cosmetic value, with stem and leaf extracts garnering attention for their abundant bioactive ingredients. Meanwhile, fermentation can enhance the effectiveness of cosmetics. The aim of this study was to optimize ginseng fermentation to produce functional cosmetics. Ginseng stem and leaf extracts were fermented with five different strains of lactic acid bacteria. Using 2,2-diphenyl-1-picrylhydrazyl (DPPH), hydroxyl radical (·OH), and superoxide anion (O_2_^·−^) scavenging activities as indicators, the fermentation process was optimized via response surface methodology. Finally, validation of the antioxidant activity of the optimized fermentation broth was performed using human skin cells (HaCaT and BJ cells). Based on the antioxidant potency composite comprehensive index, *Lactiplantibacillus plantarum* 1.140 was selected, and the optimized parameters were a fermentation time of 35.50 h, an inoculum size of 2.45%, and a temperature of 28.20 °C. Optimized fermentation boosted antioxidant activity: DPPH scavenging activity increased by 25.00%, ·OH by 94.00%, and O_2_^·−^ by 73.00%. Only the rare ginsenoside Rg_5_ showed a substantial rise in content among the 11 ginsenosides examined after fermentation. Furthermore, the flavonoid content and ·OH scavenging activity were significantly negatively correlated (*r* = −1.00, *p* < 0.05), while the Rh_1_ content and O_2_^·−^ scavenging activity were significantly positively correlated (*r* = 0.998, *p* < 0.05). Both the 0.06% (*v*/*v*) and 0.25% (*v*/*v*) concentrations of the optimized broth significantly promoted cell proliferation, and notable protective effects against oxidative damage were observed in HaCaT cells when the broth was at 0.06%. Collectively, we demonstrated that ginseng fermentation extract effectively eliminates free radicals, preventing and repairing cellular oxidative damage. This study has identified new options for the use of fermented ginseng in functional cosmetics.

## 1. Introduction

Ginseng is a perennial plant that belongs to the Araliaceae family; it contains a variety of bioactive compounds that have antioxidant, anti-aging, anti-inflammatory, and other effects [1]. Hence, ginseng is traditionally used as a raw material in the production of cosmetics in China and South Korea [2]. The bioactive compounds of ginseng include ginsenosides, polysaccharides, phenols, and flavonoids; ginsenosides are the primary active constituents, comprising approximately 4% of the total content of ginseng. They are found in abundant quantities in various parts of the ginseng plant, including the roots, stems, leaves, and fruit. Ginsenosides Rc, Re, Rb_2_, Rf, and Rb_1_ are the major ginsenosides, accounting for more than 90% of the total ginsenosides (TGs) [3]. Rare ginsenosides, such as Rg_3_, CK, Rh_2_, and F_2_, constitute a minimal proportion of the TGs, typically less than 0.1% in natural plant specimens; for example, the quality content of Rg_3_ is usually 0.0003% to 0.03%. Interestingly, these rare ginsenosides play a more significant role in the efficacy of ginseng than their more common counterparts [4], and due to their notable pharmacological activities (e.g., antioxidant, anti-aging, and anti-inflammatory effects) and market demand, there is growing interest in these ginsenosides across academia and industrial sectors. For example, ginsenoside CK can not only improve the antioxidant enzyme activity of the skin but also protect keratinocytes and dermal fibroblasts from ultraviolet damage, exhibiting a photoprotective effect [5].

Despite exhibiting stronger biological activity than the major ginsenosides, the minor ginsenosides are naturally present in relatively low quantities in ginseng, which makes it difficult to meet the needs of industrial production and market products [6]. Due to its low production cost, short cycle, few byproducts, and product safety, microbial fermentation has been widely used in traditional Chinese medicine (TCM) [7]. Numerous studies have also been conducted on the fermentation practices used in TCM. For instance, Pham [8] tested the effects of a fermented black ginseng (FBG) product on human skin fibroblasts (HS68) and found that it had potential antioxidant activity and could be used as an active ingredient in cosmetics due to its anti-wrinkle activity. Also, there is increasing interest in the fermentation of ginseng using probiotics [9,10]. Probiotics have been shown to contribute to the fermentation and transformation of the saponins in ginseng in a highly specific and safe manner, and high yields have been obtained; hence, their use has become one of the important ways to improve the biological activity of ginseng products [11,12]. Lactic acid bacteria (LAB) are well-known probiotics, and LAB fermentation is recognized by the China Food and Drug Administration (CFDA) as a safe method for use in food production. Given that the transformation of ginsenosides using LAB does not require the addition of chemical reagents that may cause harm, does not generate pollution, and exhibits good sustainability [13], it is considered a green and environmentally friendly process.

The skin forms the human body’s main barrier, and its function is primarily mediated by the structure of the epidermis and dermis [14]. The epidermis, which is the outermost layer of the skin, is mainly composed of keratinocytes and is vulnerable to various types of oxidative stress damage. The dermis is chiefly composed of human skin fibroblasts and a matrix of collagen-rich fibers that the fibroblasts produce (by taking in essential amino acids) to reduce damage caused by external factors [15]. Oxidative stress can easily trigger the oxidation of proteins and cell membrane lipids, DNA damage, and other processes in the skin [16] that lead to accelerated skin aging, wrinkle formation, and reduced elasticity [17].

With more than 2000 years use history, Ginseng is believed to maintain vigor and vitality in the ancient Chinese Meteria Medica ShenNongBenCaoJing (Patron of Agriculture’s Herbal Classic) [18]. It exhibits strong antioxidant activity, has been shown to eliminate oxygen free radicals, has anti-aging effects [19], and plays a significant protective and reparative role in skin damaged by oxidation [20]. Research has revealed that ginseng stems and leaves, like the roots, contain active components and that the content is three times higher in these plant parts, with the saponin concentration ranging from 7.6% to 12.6% [21]. The antioxidant activity of the stems and leaves also surpasses that of the roots [22]. However, despite the superior attributes of the stem and leaf material, ginseng stem and leaf resources are associated with substantial waste [23]. This underlines the need for better utilization of ginseng stem and leaf material.

Recently, we conducted several studies on TCM fermentation. Notably, the two-way fermentation of *Schizophyllum commune Fr.* and *Radix Puerariae* significantly increased the yield and antioxidant activity of produced exopolysaccharides [24], and these metabolites were found to prolong the life and improve the health of *Caenorhabditis elegans* [25]. In another study, we analyzed the active substances in the fermentation broth of *Tremella albophyllum* and found that they had antioxidant activity [26]. Additionally, *Lactobacillus acidophilus* was employed to ferment dandelion, and the capacity of the fermented extract to reduce uric acid and the underlying mechanism were validated [27]. Our previous studies have included various attempts to realize the bidirectional fermentation of probiotics and medicinal plants [24,25,26,27]. Typically, a cosmetic ingredient named SPG (Schizophyllan), has been put into commercial production by MARUBI, a listed company based in Guangzhou, China. Currently, we have explored the utilization of ginseng stems and leaves to produce more active potential cosmetic ingredients. Meanwhile, the optimized fermentation process results in a significantly shortened duration of 35.5 h, which is highly advantageous for industrial applications.

This study was aimed (i) to investigate the efficacy of a ginseng fermentation broth produced from stems and leaves as a functional ingredient in antioxidant cosmetics and (ii) to optimize the utilization of ginseng stem and leaf material. To achieve these aims, we utilized a ginseng stem and leaf extract as the raw material and used *Lactiplantibacillus plantarum* to optimize fermentation. We explored the antioxidant activity of products generated under different conditions and further optimized the antioxidant activity of the fermentation broth through response surface methodology. An oxidative damage cellular model was employed to determine the antioxidant potential of the fermentation broth in an environment that reflected the body’s actual metabolism. The findings of this study may aid the development of protective and reparative cosmetics that include ginseng fermentation products with anti-aging effects.

## 2. Results and Discussion

### 2.1. Antioxidant and β-Glucosidase Activities of the Lactobacillaceae Strains

**Figure 1:**
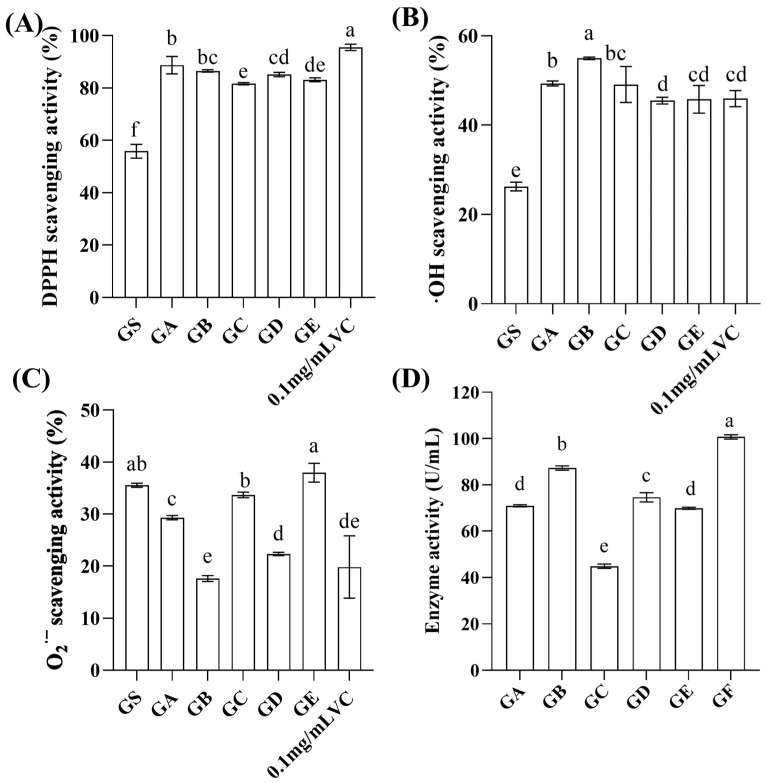
Antioxidant and β-glucosidase activities of the GA, GB, GC, GD, and GE fermentations. (**A**) DPPH scavenging activity; (**B**) ·OH radical scavenging activity; (**C**) O_2_^·−^ radical scavenging activity; (**D**) β-glucosidase activity; Data are expressed as mean ± SD, *n* = 3; values with no letters in common are significantly different (*p* < 0.05).

The antioxidant activity of the GS and five *Lactobacillus* fermentation broths (Figure 1A–C) was analyzed using three different assays that each evaluated the DPPH, ·OH, and O_2_^·−^ scavenging capacity. Compared with the GS, the five *Lactobacillus* fermentation broths had significantly (*p* < 0.05) superior antioxidant properties in terms of their capacity to scavenge DPPH and ·OH. Moreover, the samples had greater activity against DPPH compared to ·OH and O_2_^·−^, as shown by the respective scavenging rates of 81.65–88.71%, 45.48–54.96%, and 17.63–33.68%. Lee et al. [28] found that fermentation can improve the antioxidant activity of ginseng when studying the antioxidant effect of fermentation on ginseng, which is consistent with the results of this experiment. Xu et al. [29] found that LAB can produce certain active substances during metabolism, which efficiently scavenge free radicals extracellularly. This may explain the increase in antioxidant activity observed as a result of ginseng fermentation.

When the DPPH scavenging activity of the five fermentation broths (diluted 20 times) was analyzed, it was found that the GA fermentation broth showed higher activity than the GC, GD, and GE fermentation broths; however, the difference between the capacities of the GB and GA fermentation broths was not statistically significant (*p* > 0.05). The 0.1 mg/mL VC solution exhibited the highest DPPH scavenging capacity (95.48 ± 0.99%), with the GB fermentation broth demonstrating a scavenging rate merely 9.43% lower than that of the 0.1 mg/mL VC solution. Among the five fermentation broths, the GB fermentation broth had the most significantly improved (*p* < 0.05) ·OH scavenging activity; it was 109.37% and 19.71% higher than the GS and 0.1 mg/mL VC solutions, respectively. In terms of the capacity to scavenge O_2_^·−^, the GA, GC, GD, and GE fermentation broths were found to have low scavenging activity (22.33–37.97%) but still higher than that of the 0.1 mg/mL VC solution (19.82 ± 4.88%). It is interesting that the GB fermentation broth, which had significant DPPH and ·OH radical inhibitory activity, showed the lowest scavenging O_2_^·−^ capacity. Different free radical scavenging assays showed different antioxidant activity, which might be due to variations in the content and types of antioxidants present in fermentation broths from different strains.

Li et al. [30] found that LAB can produce β-glucosidase during the metabolic process, and the magnitude of its activity is the main factor determining the conversion of saponins into rare saponins. β-Glucosidase can also utilize substrates to generate other active substances, thereby enhancing the antioxidant activity of the fermentation broth [31]. Figure 1D shows that the order of β-glucosidase enzyme activity from highest to lowest was GF > GB > GD > GA, GE > GC. The GB fermentation broth had the highest β-glucosidase activity (87.30 U/mL) among the five types of broths. This indicates that the β-glucosidase produced by GB metabolism can better cleave the glycosidic bonds of substrates, releasing more active substances.

Therefore, considering the capacities of the fermentation broths to scavenge the three types of free radicals and their β-glucosidase activities, GB was selected for the subsequent fermentation of the ginseng extract and the associated experiments.

### 2.2. Preliminary Screening for Culture Conditions

#### 2.2.1. Effect of Fermentation Time on Capacity to Scavenge Radicals

**Figure 2:**
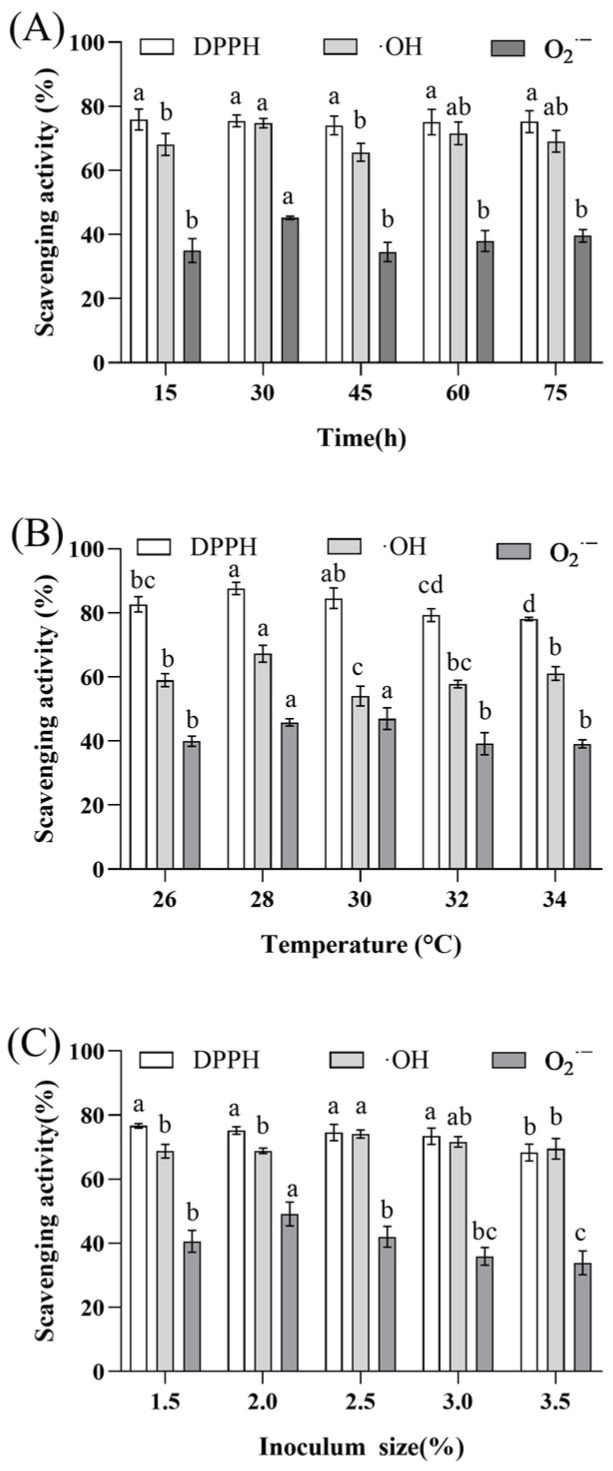
Antioxidant activity of fermentation broth under different fermentation times, fermentation temperatures, and inoculum sizes. (**A**) fermentation times; (**B**) fermentation temperatures; (**C**) inoculum sizes. Data are expressed as mean ± SD, *n* = 3. Values with no letters in common are significantly different (*p* < 0.05).

The fermentation time not only affects the growth state of the microorganism but also influences the antioxidant activity of the fermentation product [32], and determining the fermentation endpoint is critical to improving the industrial production capacity and economic benefits of fermentation. As shown in Figure 2A, changing the fermentation time had no significant effect on the radical scavenging rates, which peaked at 30 h. The fermentation broth samples showed a mean ·OH clearance of 74.77 ± 1.19%, which was 35.32% higher than that of the non-fermented samples. The mean O_2_^·−^ clearance in the fermentation broth samples was 45.23 ± 0.43%, an increase of 16.97% compared to that in the non-fermented samples. After comprehensively analyzing the effect of fermenting ginseng in the presence of *L. plantarum* for different fermentation times on the antioxidant activity, 15–45 h was selected for further response surface analysis.

#### 2.2.2. Effect of Fermentation Temperature on Capacity to Scavenge Radicals

Temperature mainly affects microbial metabolism—and hence fermentation—via its influence on enzyme production and activity; therefore, the temperature must be carefully controlled during fermentation to achieve the optimal outcome [33]. As shown in Figure 2B, in the temperature range of 26–34 °C, as the temperature increased, the DPPH and O_2_^·−^ scavenging rates of the fermentation broth showed an increasing and then a decreasing trend. The overall ·OH scavenging rate showed a similar trend, with the lowest value (54.04 ± 2.49%) recorded at 30 °C. The DPPH scavenging rate peaked at 28 °C (87.61 ± 1.57%), as did the ·OH scavenging rate (67.27 ± 2.14%), while the O_2_^·−^ scavenging rate peaked at 30 °C (47.00 ± 2.73%).

#### 2.2.3. Effect of Inoculum Size on Capacity to Scavenge Radicals

In the fermentation process, the inoculum size is an important factor that affects the growth and reproduction of fermentation strains [34]; hence, determining the microbial inoculum size is vital for optimizing industrial production. As shown in Figure 2C, when the inoculum size ranged from 1.5% to 3.5%, the ·OH and O_2_^·−^ scavenging capacity first increased and then decreased. The ·OH scavenging capacity peaked at 74.12 ± 0.99% when the inoculum size was 2.5%. The O_2_^·−^ scavenging capacity peaked at 49.17 ± 2.99% when the inoculum size was 2.0%. In terms of DPPH scavenging, a significant reduction in DPPH was observed when the inoculum size was 3.5%; however, the inoculum size did not have any other significant effects on DPPH scavenging. The antioxidant activity of the fermentation broth samples was highest in the range of 2.0–3.0%; thus, an inoculum size of 2.0–3.0% was selected for further analysis.

### 2.3. Response Surface Optimization

#### 2.3.1. Box–Behnken Experimental Design and Model Fitting

Response surface analysis based on the Box–Behnken design is a statistical and mathematical method used to reflect the optimal corresponding conditions obtained when the interactions between various factors in a multi-factor system reach the maximum response [35,36]. The Box–Behnken experimental design and results are shown in Table 1. Design-Expert 11 software was used to fit the data shown in Table 1 and the DPPH, ·OH, and O_2_^·−^ scavenging values as response values to multiple regression equations, and the following quadratic multinomial regression equations were obtained:

(1) $$\mathrm{DPPH}\;\mathrm{scavenging}\;\mathrm{activity}\;\%=-69.20346+0.299786A\;-\;14.04878B\;-30.97059C\;+0.039120\mathrm{AB}\;-\;0.095927\mathrm{AC}+0.188227\mathrm{BC}\;-\;{0.024671A}^{2}-\;{0.288259B}^{2}-\;{5.65562C}^{2}$$

(2) $$\cdot \mathrm{OH}\;\mathrm{scavenging}\;\mathrm{activity}\;\%=-270.88194+3.62171A+17.26828B+48.43020C\;-\;0.088658\mathrm{AB}\;-\;0.178723\mathrm{AC}+0.275307\mathrm{BC}\;-\;{0.013660A}^{2}-\;{0.280090B}^{2}-\;{10.22727C}^{2}$$

(3) $${O_{2}}^{\cdot -}\mathrm{scavenging}\;\mathrm{activity}\;\%=-589.96181+2.67379A+34.39914B+85.38185C\;-\;0.044285\mathrm{AB}\;-\;0.167331\mathrm{AC}\;-\;0.279052\mathrm{BC}\;-\;{0.014265A}^{2}-\;{0.569831B}^{2}-\;{14.55579C}^{2}$$

where A represents fermentation time, B represents fermentation temperature, and C represents inoculum size.

For the model with the DPPH scavenging rate as the response value, only one term (A) was significant (*p* < 0.05), while the effects of B and C were not significant (*p* > 0.05). The quadratic term A^2^ had significance (*p* < 0.05); however, the quadratic terms B^2^ and C^2^ did not reach significance, indicating that the interaction had little effect on the DPPH scavenging activity of the fermentation broth. Hence, the DPPH scavenging rate as the corresponding model was not established.

Next, for the model with the ·OH scavenging rate as the response value, the F value is 3.20 with *p* > 0.05, indicating no significance of the model equation. Usually, an R^2^ value close to 1 shows a better explanation of the variability of the experimental data by the proposed model. However, the correlation of determination R^2^ value of 0.8045 and the adjusted R^2^ value of 0.5532, indicating that the actual value was not close to the predicted value of the model. Thus, the response surface model with the ·OH scavenging rate as the response value was also deemed unsuitable for the analysis of the optimal fermentation conditions.

Finally, for the model with the O_2_^·−^ scavenging rate as the response value, it can be seen from Table 2 that there is a significant regression with a *p*-value of 0.001 < 0.05. The *p*-value (0.7682) of lack-of-fit indicated insignificant lack-of-fit relative to pure error, hence the model equations were satisfactory for the prediction of O_2_^·−^scavenging activity in the whole experimental run. The goodness of fit of the regression model was evaluated with the correlation of determination R^2^ value of 0.9483. Adjusted R^2^ value of 0.8818, indicating that the actual value was also close to the predicted value of the model. Therefore, these results implied that the model had high credibility and could be used to predict changes in the antioxidant activity of the GB fermentation broth.

The terms A, A^2^, B^2^, and C^2^ were found to be significant (*p* < 0.05), while the effects of AB, AC, and BC on the response values were not significant (*p* > 0.05). The larger the *F*-value, the greater the influence of this factor on the O_2_^·−^ scavenging rate. Based on the *F*-value results, the influencing factors that affected the O_2_^·−^ scavenging activity of the fermentation broth were ranked as follows: A > B > C. Hence, we deduced that the O_2_^·−^ scavenging rate is a better choice for to determine the optimal fermentation conditions.

#### 2.3.2. Response Surface and Contour Plot Analysis

**Figure 3:**
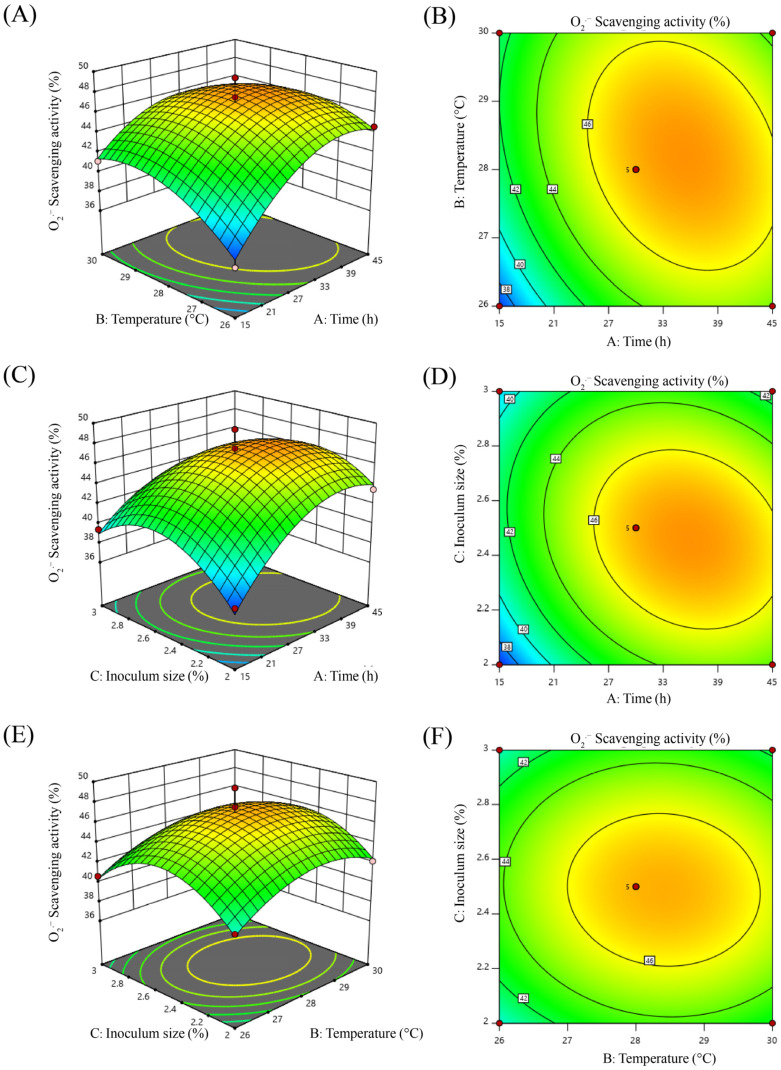
Response surface and interaction plan of each factor with O_2_^·−^ radical scavenging rate as the response value. (**A**,**B**) 3D and contour plots of the interaction between fermentation time and fermentation temperature, (**C**,**D**) 3D and contour plots of the interaction between fermentation time and inoculum size, (**E**,**F**) 3D and contour plots of the interaction between fermentation temperature and inoculum size. (**A**) Fermentation time, (**B**) fermentation temperature, (**C**) inoculum size.

Response surface models intuitively and accurately depict the interaction between two factors [37,38]. As can be seen in Figure 3A,C,E, in our case, the response surface curves changed sharply, indicating that they had a greater influence on the response value. When the contours of the interaction of the influencing factors are oval or saddle-shaped, the interaction of the two factors is significant; it is not significant when the contours are round [26]. As shown in Figure 3B,D, the contour plot approached an oval shape, and the slope of the surface was steep, indicating that the interaction of the two groups of factors was significant; in other words, the interaction of the two factors had a significant effect on the O_2_^·−^ scavenging rate. The medium–high line in Figure 3F was also close to the ellipse, but the slope of the surface was relatively flat, and thus the interaction was relatively insignificant; that is, the effect on the response value was small. The results are consistent with the results of the regression analysis.

#### 2.3.3. Optimal Fermentation Process

**Figure 4:**
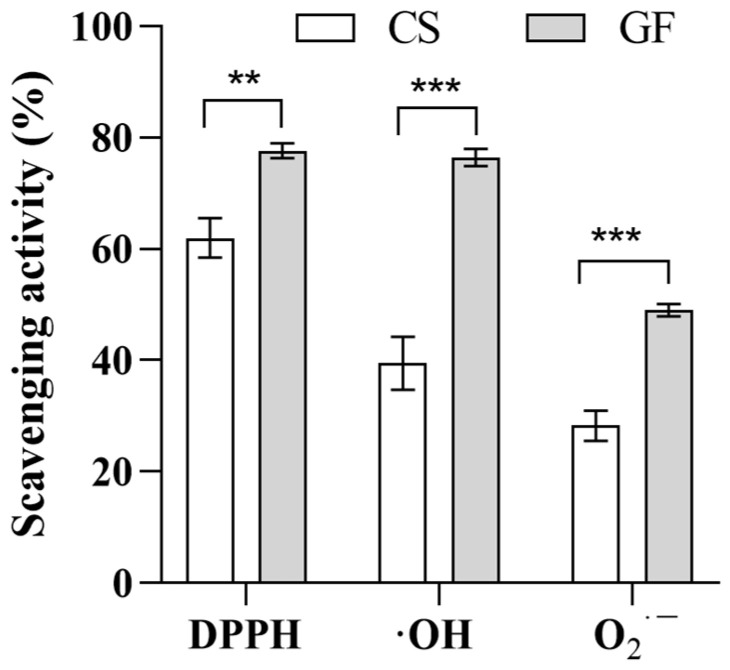
Comparison of antioxidant results of fermentation liquid outside GB under optimized conditions. ** 0.001 < *p* < 0.01, *** *p* < 0.001.

To validate the proposed model, a series of fermentations were performed in which the fermentation time, temperature, and inoculum size were varied in accordance with the response surface analysis results (Table 3). Using Equation (3), the process parameters that were found to result in optimal radical scavenging capacity were a fermentation time of 35.52 h, an inoculum size of 2.45%, and a fermentation temperature of 28.20 °C. As shown in Figure 4, the average O_2_^·−^ scavenging rate was 47.53 ± 0.55%, and this value did not significantly differ from the predicted value (*p* > 0.05). The fact that the experimental value was very close to the predicted value proved that the results were reasonable and reliable. The broth produced under these fermentation conditions exhibited a 73.00% increase in O_2_^·−^ scavenging capacity, a 94.00% increase in ·OH scavenging capacity, and a 25.00% increase in the DPPH scavenging rate compared to the pre-fermentation solution. This approach not only significantly (*p* < 0.05) increased the antioxidant activity but also improved the fermentation efficiency.

### 2.4. Correlation Analysis of the Active Ingredients and Antioxidant Activity of the Optimized Broth (GF)

#### 2.4.1. Active Ingredients

**Figure 5:**
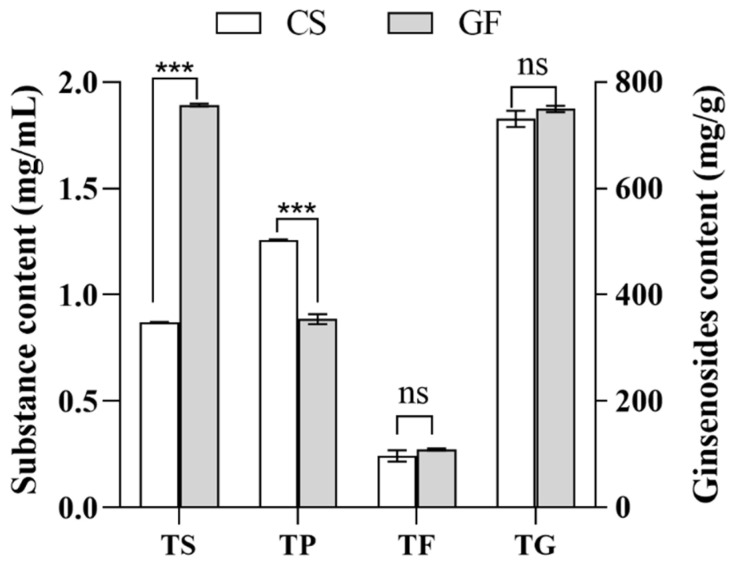
Total sugars, total phenol, total flavonoids, and total ginsenosides in fermentation broth under optimized conditions. ns: *p* > 0.05, *** *p* < 0.001.

It is well-known that fermentation broths are complex systems and that fermentation with LAB can produce β-glucosidase. This enzyme breaks the glycosidic bonds in ginsenosides, phenolic flavonoids, and other compounds, leading to structural changes in many active ingredients and the potential formation of new substances [31]. As shown in Figure 5, we compared the TS, TP, TF, and TG contents of the optimized fermented broth (GF) with those of the unfermented solution (GS). We found that the TS content of the GF (1.89 ± 0.01 mg/mL) was significantly (*p* < 0.05) higher than that of the GS, showing an increase of 117.00%. The TP content of the GF was significantly (*p* < 0.05) lower than that of the GS, showing a decrease of 29.00%. Additionally, both the TF content (0.27 ± 0.00 mg/mL) and the TG content (749.72 ± 4.74 mg/mL) of the GF did not significantly differ from those of the GS (*p* > 0.05).

In contrast to some previous studies, such as that conducted by Wang et al. [39], fermentation did not result in a significant increase in the TP content in the current study, but instead a notable decrease. Concurrently, fermentation resulted in a significant increase in the TS content in this study. We speculate that this difference may be attributed to the fermentation process causing the breakdown of high-molecular-weight phenolic compounds, leading to the cleavage of glycosidic bonds. This process releases sugar molecules and low-molecular-weight active substances with hydroxyl groups. Thus, although the phenolic content decreased, the increase in the TS content may have been due to sugar molecules being released by phenolics and the degradation of insoluble high molecular weight polysaccharides into soluble polysaccharides with relatively low molecular weights [40].

Flavonoids are also a type of phenolic substance, and the lack of a significant change in their content during the fermentation process may have been due to the expression of β-glucosidase by the *Lactobacillus* strains. This enzyme may have catalyzed the conversion of bound flavonoids in the fermentation liquid into free-form flavonoids [39], which would have resulted in a relatively constant level of flavonoids in the fermentation liquid. This result is similar to that of Xu et al. [29].

Many studies have shown that the TG content can be significantly reduced by microbial transformation and that the main ginsenosides can produce many rare ginsenosides [41,42]. In this study, there was no significant difference between the saponin content before and after fermentation, and these results reflected those reported by Lee [43]. The reason for this may be that the ligase produced by the bacterial strain reconnected the glycosides in ginseng and those hydrolyzed by enzymes, such as β-glucosidase, into new monomeric ginsenosides, resulting in no change in the TG content. Alternatively, the enzymatic activity that occurred during fermentation caused changes in the sugars or other components of ginseng, which resulted in the formation of ginsenosides or ginsenoside components through a series of complex reactions. However, further experiments and analyses are required to confirm that the developed fermentation process results in no change in the TG content and the mechanism(s) responsible for the observed findings.

#### 2.4.2. Ginsenoside Monomer Content

**Figure 6:**
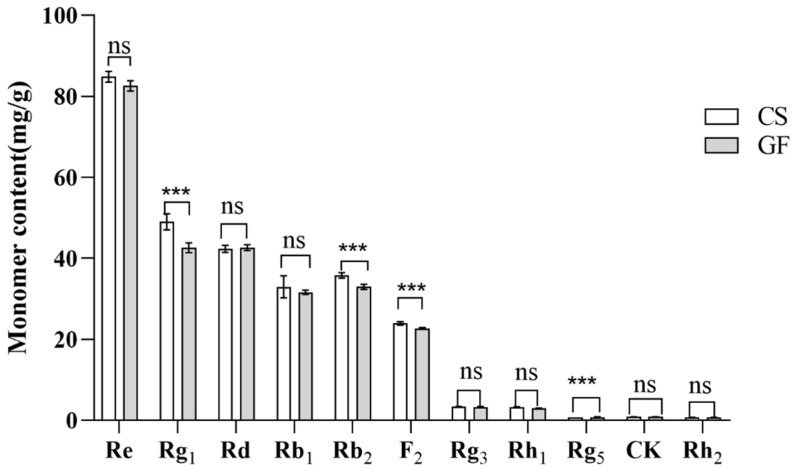
Conversion of ginsenoside monomer components under optimized conditions. ns: *p* > 0.05, *** *p* < 0.001.

The 11 ginsenosides are the main active components of ginseng, and the rare ginsenosides have potential application value [44]. Thus, the concentrations of the 11 ginsenosides in the ginseng solution before and after fermentation under the optimized conditions were determined (Figure 6). The concentrations of the major ginsenosides (Re, Rg_1_, Rd, Rb_1_, and Rb_2_) were higher than 30.00 mg/g. The concentrations of the rare ginsenosides (Rg_3_, Rh_1_, Rg_5_, CK, and Rh_2_), except F_2_, were lower than 3.50 mg/g. After fermentation, the Rg_1_, Rb_2_, and F_2_ contents were significantly lower, decreasing by 13.10%, 7.8%, and 5.30%, respectively (*p* > 0.05), and the Rg_5_ content was significantly higher, increasing by 9.20% (*p* > 0.05). The F_2_ concentration in the ginseng raw material was high (up to 23.90 mg/g), which may have been due to the conversion of the ginseng extract powder during the ultrasonic extraction steps.

The reason for the changes in the concentration and type of saponins was that glucose groups at the C_6_ and C_20_ positions of the protopanaxatriol (PPT) saponin and the C_3_ and C_20_ positions of the ginsenosides of the protopanaxadiol saponin were hydrolyzed during the fermentation process, resulting in rare ginsenosides [45]. Palaniyandi et al. [9] showed that Rb_1_/Rb_2_ glycol saponins are transformed by microorganisms to generate Rd, and Rd can then be further converted to Rg_3_, Rh_2_, F_2_, and CK. Bai [46] and Tran [47] speculated that the conversion pathways of the diol ginsenosides are Rb_1_/Rb_2_ → Rd→Rg_3_ → Rh_2_, Rb_1_/Rc → Rd → Rg_1_, Rg_3_ → Rg_5_ + Rk_1_, and Rb_1_ → Rd → F_2_ → Rg_3_ → CK, while the triol ginsenoside conversion pathway is Re → Rg_1_ → Rh_1_ → Rh_4_ → PPT.

In this study, the Rb_2_ content decreased, while the Rd, Rg_3_, Rh_2_, and CK contents did not change significantly (*p* > 0.05). Only the Rg_5_ content increased significantly (*p* < 0.05). Thus, it can be speculated that Rd, Rg_3_, Rh_2_, and CK may have been intermediate or end products that were generated and consumed at the same time, resulting in the observed decrease in Rb_2_ content and increase in Rg_5_ content. In the case where the content of Rg1 decreases while Rh_1_ remains unchanged, it is possible that Rh4 or PPT, along with other unidentified and quantitatively undetermined saponins, have been produced (Figure S1). The high concentration of saponin substrates can affect the hydrolytic ability of glycosidases towards saponin glycosidic bonds [48]. Thus, apart from Rg_5_, there was no significant changes in the content of detected rare ginsenosides before and after fermentation. To gain a more comprehensive understanding of the types of ginsenosides and other components present in the fermentation broth, further qualitative and quantitative studies are necessary, including studies that incorporate untargeted and targeted metabolomics approaches.

#### 2.4.3. Correlation Analysis

Ginseng has the capacity to scavenge free radicals because it contains polysaccharides [49], polyphenols [50], flavonoids, ginsenosides [51], and other active ingredients [52]. To identify the components of the fermentation broth responsible for its capacity to scavenge the three types of free radicals, a correlation analysis was conducted using the data on the various active ingredients and the DPPH, ·OH, and O_2_^·−^ scavenging activities of the GF (Table 4). A significant negative correlation was observed only between flavonoids and ·OH scavenging activity (*r* = −1.00, *p* < 0.05). This result is similar to that of Huang et al. [53], where the flavonoid components were negatively correlated with the free radical scavenging capacity. This phenomenon may be attributed to the enzymatic breakdown of flavonoids by β-glucosidase, which may have generated trace amounts of components with enhanced antioxidant activity.

Even though no significant positive correlations were found between the four major types of active components, especially the significantly increased polysaccharides, and the three types of free-radical scavenging activities, the findings do not imply that these components had no impact on the antioxidant activity of the fermentation broth. Other low-molecular-weight active components within the major categories may still have played a role in the antioxidant activity. For instance, the TG content showed no significant correlation with the three free-radical scavenging rates. However, among the 11 ginsenosides, Rh_1_ exhibited a significant correlation with the O_2_^·−^ scavenging activity (Table S1), with a correlation coefficient of 0.998. This is similar to the findings of Liu et al. [54], who reported that G-Rh_1_ acts as a pro-oxidant and synergistically exhibits antioxidant properties with α-tocopherol.

In addition to the potential involvement of other low-molecular-weight substances, it is also plausible that many of the active components were working in a synergistic manner. Therefore, it is necessary to conduct further studies to identify both the components and the ways in which they contribute to the antioxidant activity of the fermentation broth.

#### 2.4.4. Effects of the GS and GF on the Activity of HaCaT and BJ Cells

To further evaluate the in vitro biological activity of the GS and GF, we used two human cell lines, HaCaT (keratinocytes) and BJ (fibroblasts), which are commonly used to evaluate the effects of antioxidant compounds on skin [55,56]. Specifically, we conducted cell viability assays to determine the effect of the GS and GF on the growth of HaCaT and BJ cells.

**Figure 7:**
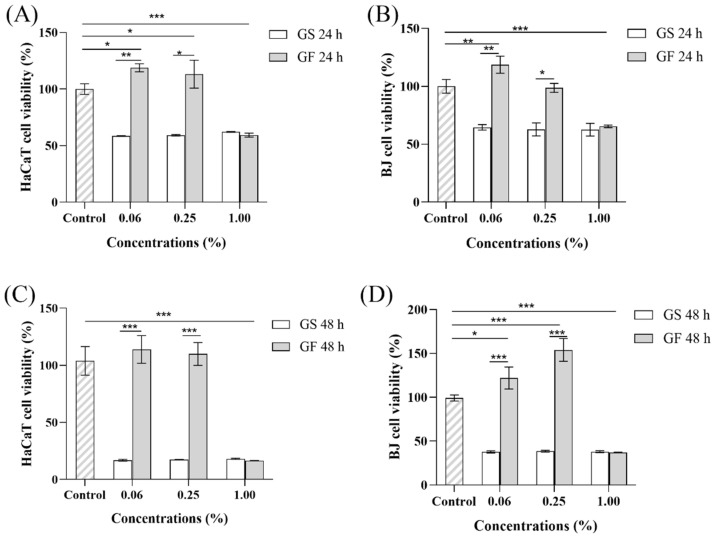
Fermentation broth fermented under different volume fraction optimization conditions—HaCaT/BJ cell activity. (**A**) Fermentation broth under optimal conditions administered to HaCaT cells for 24 h; (**B**) fermentation broth administered to BJ cells under optimal conditions for 24 h; (**C**) fermentation broth administered to HaCaT cells for 48 h under optimal conditions; (**D**) fermentation broth administered to BJ cells under optimal conditions for 48 h. * *p* < 0.05, ** 0.001 < *p* < 0.01, *** *p* < 0.001.

In the HaCaT cells, the cell viability decreased as the concentration of the GF increased after 24 and 48 h of exposure (Figure 7A,C). At 0.06% GF, the cell viability was 110.00% at both time points. However, at 1.00% GF, the cell viability decreased to 59.36% after 24 h and dropped further to 16.39% after 48 h. When HaCaT cells were exposed to three different GS concentrations, the cell viability was around 60.00%, and no significant differences were observed. With prolonged exposure, the cell viability decreased to approximately 18.00%. According to the international standard ISO 10993-5 (2009) [57], a reduction in cell viability of more than 30.00% indicates cytotoxic effects. Thus, it is evident that 1.00% GF and various concentrations of GS had significant cytotoxic effects on the HaCaT cells, and this cytotoxicity intensified with prolonged exposure. The underlying reason for this phenomenon may be that increasing the fermentation broth concentration and the duration of exposure led to increases in the levels of the cytotoxic substances and their effects and that the cytotoxic effects eventually overwhelmed the protective and reparative effects of the active ingredients [58].

In the BJ cells (Figure 7B,D), the viability of cells exposed to the GF closely mirrored that observed in the HaCaT cells. However, a notable difference emerged at 0.25% GF: the cell viability after 48 h of exposure was significantly higher (*p* < 0.05) than that after 24 h, exhibiting an increase of 55.84%. Notably, 1.00% GF had a greater impact on the cell viability of BJ cells compared to HaCaT cells. In the HaCaT cells, a significant reduction was observed only at 1.00% GF; there were no significant changes at the other two concentrations. When BJ cells were exposed to the GS, the dosing trend and cytotoxicity pattern also resembled those observed in the HaCaT cells. However, at 48 h, the cell viability was higher in the BJ cells than in the HaCaT cells, at approximately 210.40% of the HaCaT cell viability.

From these findings on the impact of the GS and GF on cell growth in HaCaT and BJ cells, it is evident that subjecting the GS to the optimized fermentation conditions significantly reduced its cytotoxicity (*p* < 0.05). Below a concentration of 0.25%, the GS exhibited no cytotoxicity and even enhanced cell viability. This phenomenon may be attributed to harmful components in the GS being reduced in terms of their activity during the *Lactobacillus* fermentation, which mitigated the toxicity. This finding aligns with the results reported by Wei et al. [59]. Moreover, at low concentrations (0.06–0.25%), the GF promoted cell proliferation, suggesting a potential positive effect on skin cells. This may have been due to the presence of certain substances in the fermentation broth that facilitated cell growth. At extremely low concentrations, these substances may promote cell growth. However, they may inhibit cell growth or cause cell death at high concentrations [60].

#### 2.4.5. Effects of the GS and GF on HaCaT and BJ Cells in an Oxidative Stress Model

H_2_O_2_ has long been widely used to establish cellular models of acute oxidative stress for investigating free radical-induced cell damage [61]. To confirm a suitable H_2_O_2_ concentration for our in vitro H_2_O_2_-induced injury model, HaCaT cells were exposed to nominal concentrations of H_2_O_2_, and their viability was measured using the CCK-8 assay. As shown in Figure S2, 650 µM H_2_O_2_ caused about 50.00% cell mortality. Following Zhang et al. [62], who considered the concentration of H_2_O_2_ that led to 50.00% cell death as the optimal damage condition, 650 µM H_2_O_2_ was selected as the optimal concentration for inducing oxidative stress for use in all subsequent experiments.

**Figure 8:**
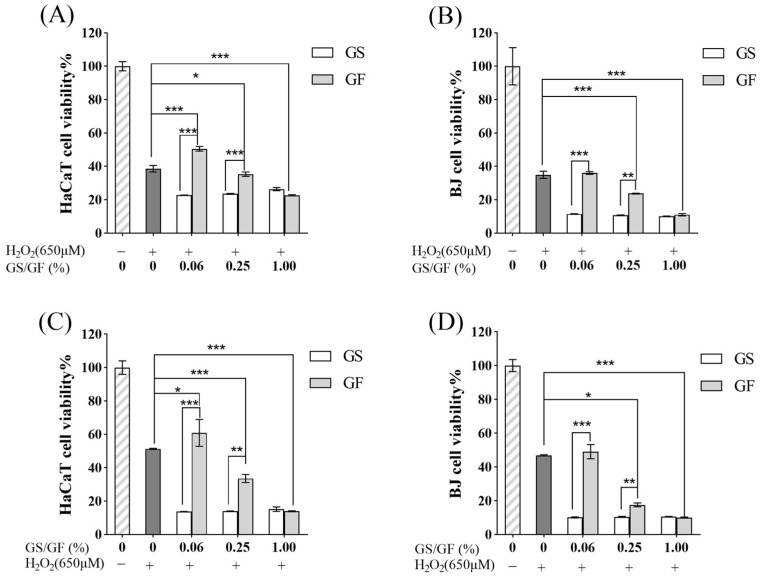
Effects of GS and GF fermentation broths on the activity of H_2_O_2_-treated HaCaT/BJ cells. (**A**,**B**) The reparative effect of H_2_O_2_ pretreatment on H_2_O_2_-treated HaCaT and BJ cells damage; (**C**,**D**) The protective effect of GS and GF pretreatment on H_2_O_2_-treated HaCaT and BJ cells damage. * *p* < 0.05, ** 0.001 < *p* < 0.01, *** *p* < 0.001.

HaCaT and BJ cells with H_2_O_2_-induced oxidative stress were incubated with different concentrations of the GS and GF (Figure 8A,B). In cells that were exposed to H_2_O_2_ but not the GS or GF, the cell viability was 38.63% in HaCaT cells and 34.97% in BJ cells. After exposure to H_2_O_2_, cells are irreversibly damaged [63]. This was observed when cells were exposed to H_2_O_2_ before the medium containing H_2_O_2_ was replaced with normal medium and the cells were incubated for 24 h; the cell viability was still reduced. As shown in Figure 8A, when the GF concentration was 0.06%, the cell viability of H_2_O_2_-treated HaCaT cells was 50.56%, which was 11.93% higher than that of the control group (*p* > 0.05). This indicated that there may have been a damage-repair effect. As the GF concentration increased, the cell viability of the H_2_O_2_-treated HaCaT cells decreased in a dose-dependent manner. When the H_2_O_2_-treated HaCaT cells were incubated with different concentrations of the GS, the cell viability was lower than that of the control group. Figure 7 shows that 1.00% GF and different concentrations of GS had a cytotoxic effect on normal cultured HaCaT cells. When the effect of the GS and GF on H_2_O_2_-treated BJ cells was examined, an unexpected result emerged: H_2_O_2_-treated BJ cells incubated with different concentrations of the GS and GF did not show improved cell viability compared with the control group (Figure 8B). This suggested that neither the GS nor the GF had a damage-repair effect on the H_2_O_2_-treated BJ cells.

A different methodology was then utilized to assess the effects of the GS and GF on H_2_O_2_-treated HaCaT and BJ cells. Cells were first incubated with different concentrations of the GS or the GF for 24 h, and then H_2_O_2_ was used to induce oxidative stress (Figure 8C,D). The results were similar to those shown in Figure 8A,B. At 0.06% GF, the cell viability of the H_2_O_2_-treated HaCaT cells was 60.88%, which was 9.56% higher than the 51.32% viability of the control group (*p* < 0.05). At the other tested GF and GS concentrations, the cell viability was somewhat reduced (Figure 8C). These results may indicate that 0.06% GF provided protection against oxidative stress-related injury in HaCaT cells. Additionally, incubation with the GF or GS at different concentrations did not affect the viability of H_2_O_2_-treated BJ cells (Figure 8D), which may indicate that neither the GS nor GF provided protection against oxidative stress-related injury in BJ cells.

The above findings indicate that 0.06% GF had the capacity to repair oxidative stress-related injury or prevent oxidative stress in H_2_O_2_-treated HaCaT cells and that such a solution may contain factors that could protect the skin against aging caused by oxidative stress. However, the specific mechanism(s) utilized by the GF to repair damage or prevent oxidative stress injury must be elucidated. The preventive and reparative effects of the fermentation broth on cellular oxidative damage may involve enhancing the activity of antioxidant enzymes such as superoxide dismutase (SOD) and glutathione peroxidase (GSH-Px), inhibiting the level of lipid peroxidation product malondialdehyde (MDA) and modulating various signaling pathways, including Keap1/Nrf2/ARE, PI3K/Akt, and Wnt [64,65]. To determine the specific mechanism by which ginseng fermentation broth repairs cellular oxidative stress damage, further molecular experiments are needed for confirmation.

## 3. Materials and Methods

### 3.1. Materials, Chemical Reagents, and Instruments

Ginseng extract was purchased from Shanxi Yueda Tianrun Co., Ltd. (Weinan, China). *L. acidophilus* (GDMCC1.412), *L. plantarum* 1.140 (GDMCC1.140), *Lactobacillus casei* (GDMCC1.159), *Lactobacillus rhamnosus* (ATCC7469), and *L. plantarum* 1.648 (GDMCC1.648) were purchased from Guangdong Microbial Culture Collection Center (Guangzhou, China). Ginsenosides Re, Rd, Rg_3_, Rb_1_, CK, F_2_, Rg_5_, Rh_1_, Rh_2_, Rb_2_, and Rg_1_ were purchased from Chengdu Refins Biotechnology Co., Ltd. (Chengdu, China). Forinphenol and 1,1-diphenyl-2-picrylhydrazyl (DPPH) were purchased from Shanghai Macklin Biochemical Technology Co., Ltd. (Shanghai, China). Gallic acid and rutin were purchased from Yuanye Biotechnology Co., Ltd. (Shanghai, China). Sodium carbonate, salicylic acid, glucose, sodium chloride, and ferrous sulfate were purchased from Zhiyuan Chemical Reagent Co., Ltd. (Tianjin, China). Sodium hydroxide, aluminum nitrate, sodium nitrite, and phenol were purchased from Tianjin Damao Chemical Reagent Factory (Tianjin, China). MRS broth was purchased from Guangzhou Huankai Microbial Technology Co., Ltd. (Guangzhou, China). Chromatographic-grade methanol and acetonitrile were purchased from Guangzhou Guanglianjin Chemical Co., Ltd. (Guangzhou, China).

HaCaT and BJ cell lines were obtained from Guangdong Marubi Biotechnology Co., Ltd. (Guangzhou, China). High-glucose Dulbecco’s Modified Eagle Medium (DMEM) and trypsin were obtained from Gibco (Grand Island, NY, USA). Fetal bovine serum was obtained from Hangzhou Sijiqing Bioengineering Materials Co., Ltd. (Hangzhou, China).

The following equipment was used for the experiment: portable pressure steam sterilizer, Shanghai Lichengbangxi Instrument Technology Co., Ltd. (Shanghai, China); electric constant temperature incubator, Ningbo Jiangnan Instrument Factory (Ningbo, China); HC-3026R high-speed refrigerated centrifuge, Anhui Zhongke Zhongjia Scientific Instrument Co., Ltd. (Hefei, China); ELX800 microplate reader, Biotek, (Phoenix, AZ, USA); electronic constant-temperature stainless steel water bath, Shanghai Yichang Instrument Sieve Factory (Shanghai, China); KQ5200E ultrasonic cleaner, Kunshan Ultrasonic Instrument Co., Ltd. (Suzhou, China); QUINTIX125D-1CN analytical balance with an accuracy of one hundred thousandth, Beijing SciTrolley Instrument System Co., Ltd. (Beijing, China); Shimadzu LC-40D X3plus quadrupole pump, Shimadzu SIL-40C X3 automatic sampler, Shimadzu CTO-40S column oven, and Shimadzu LCMS-8045 triple quadrupole mass spectrometer, Shimadzu Corporation, (Tokyo, Japan); carbon dioxide incubator, Thermo Fisher Scientific, (Waltham, MA, USA).

In this paper, the following letter codes are used to refer to the bacterial strains: GA for *L. acidophilus*, GB for *L. plantarum* 1.140, GC for *L. casei*, GD for *L. rhamnosus*, and GE for *L. plantarum* 1.648. The optimized fermentation broth produced using GB is referred to as “GF”, and the unfermented ginseng stock as “GS”.

### 3.2. Ginseng Fermentation

#### 3.2.1. Strain Activation

The five *Lactobacillus* species, which were stored in glycerol at −80 °C, were rapidly thawed at 37 °C. Then, liquid MRS broth was inoculated with 50 mL aliquots of the bacteria at a 2.0% inoculum size. The strains were incubated aerobically at 30 °C in MRS broth for 36 h and subcultured thrice before conducting all experiments.

#### 3.2.2. Initial Fermentation of Ginseng

The ginseng extract was prepared by weighing out 8.33 g of ginseng extract powder and thoroughly mixing it with 50 mL of distilled water. Then, 0.5 g/mL glucose and 0.5 g/mL tryptone solutions were prepared and sterilized at 121 °C in an autoclave for 20 min.

Next, 1 mL samples of the activated bacterial solutions were centrifuged at 10,000 rpm for 10 min, the supernatants were discarded, and the samples were diluted in 1 mL of normal saline. Each bacterial solution was then centrifuged again at 10,000 rpm for 10 min, the supernatants was discarded, and 1 mL of normal saline was added. The bacterial solution concentration was adjusted to 1 × 10^9^ CFU/mL. The ginseng medium was inoculated with 2.0% of this solution. The glucose and tryptone solutions were added to achieve a final concentration of 10 mg/mL. Fermentation was performed in a bacterial incubator at 30 °C for 72 h. The samples were centrifuged at 10,000 rpm for 10 min, and then the supernatants were collected and stored in a freezer at −20 °C.

### 3.3. β-Glucosidase Activity Assay

The β-glucosidase activity of the supernatants was determined using the modified method of Shin et al. [66]. Briefly, 1 mL of supernatant was added to 1 mL of 5 mM p-nitrophenyl-β-glucopyranoside (pNPG) and incubated at 37 °C for 10 min. The reaction was stopped by adding 1 mL of 1.0 M sodium carbonate (Na_2_CO_3_). The released p-nitrophenol (p-NP) was measured at 405 nm using a microplate reader. A p-NP standard curve was constructed for the quantification of β-glucosidase activity (Y = 10.12X + 0.0196, R^2^ = 0.9983). One unit (U) of enzyme activity was defined as the amount of enzyme that released 1 μmol of p-NP from the substrate per mL per min at 37 °C under assay conditions.

### 3.4. Antioxidant Activity Assays

#### 3.4.1. 2,2-Diphenyl-1-picrylhydrazyl Scavenging Activity

The DPPH scavenging activity in both the GS and fermentation samples was determined as described by Chen et al. [67], with slight modifications. A 0.1 mM DPPH solution (2 mL) solubilized in ethanol was added to 50 µL of the GS or fermented ginseng samples. The mixtures were vortexed and incubated at 25 °C for 30 min in the dark. The absorbance of the mixture was measured at 517 nm, and the DPPH scavenging activity was calculated as follows:

(4) $$\mathrm{DPPH}\;\mathrm{scavenging}\;\mathrm{activity}\;(\%)=(1\;-A_{1}/A_{0})\;\times \;100$$

where A_1_ and A_0_ are the absorbance values of the sample and control, respectively.

#### 3.4.2. Hydroxyl Radical Scavenging Activity

The hydroxyl radical (·OH) scavenging capability was measured using a previously described method, with minor modifications [68]. An FeSO_4_ solution (0.5 mL, 2 mM), a salicylic acid-ethanol solution (0.25 mL, 2 mM), the sample (0.08 mL), and H_2_O_2_ (0.25 mL, 0.1% *v*/*v*) were mixed and reacted as the Fenton system. After shaking, the mixture was incubated at 37 °C for 60 min. The absorbance was measured at 510 nm, and the ·OH scavenging activity was calculated using the following equation:

(5) $$\cdot \mathrm{OH}\;\mathrm{scavenging}\;\mathrm{activity}\;(\%)=[1\;-\;(A_{1}-\;A_{2})/A_{0}]\;\times \;100$$

where A_1_ is the absorbance of the sample solution, A_2_ is the absorbance when distilled water is used in place of the salicylic acid-ethanol solution, and A_0_ is the absorbance when distilled water is used in place of the sample solution.

#### 3.4.3. Superoxide Anion Scavenging Activity

The superoxide anion (O_2_^·−^) scavenging activity of the GS and fermentation samples was assayed using the method described by Tang et al. [69], with slight modifications. For this, 2.25 mL of Tris-HCl buffer (50 mM, pH 8.2), 0.1 mL of sample, and 0.2 mL of catechol solution (3 mM, pre-warmed at 37 °C before use) were mixed. The mixtures were vortexed and incubated at 25 °C for 5 min. Then, 7.5 μL HCl (8 mM) was added to the mixture to stop the reaction. The absorbance of the GS and fermentation samples was measured at 325 nm, and the O_2_^·−^ scavenging activity was calculated as follows:

(6) $${O_{2}}^{\cdot -}\;\mathrm{scavenging}\;\mathrm{activity}\;(\%)=[1\;-\;(A_{1}-\;A_{2})/A_{0}]\;\times \;100$$

where A_1_ is the absorbance of the sample solution, A_0_ is the absorbance when deionized water is used in place of the sample solution, and A_2_ is the absorbance when deionized water is used in place of the catechol solution.

### 3.5. Determination of the Main Active Ingredients

#### 3.5.1. Total Sugar (TS) Quantification

The fermentation supernatant was precipitated overnight with a triple volume of absolute ethanol. The dried crude polysaccharides were then dissolved with distilled water in preparation for measurement of the total sugar (TS). A standard curve for glucose (Y = 4.5198X + 0.0603, R^2^ = 0.9932) was obtained by referring to the phenol-sulfuric acid method of Chen et al. [70], and the polysaccharide content of the fermentation broth was determined.

#### 3.5.2. Total Phenol (TP) Quantification

The total phenol (TP) content was determined using the Folin–Ciocalteu colorimetric method, with slight modifications [71]. A standard curve for gallic acid (Y = 2666X + 0.0093, R^2^ = 0.9965) was prepared to determine the TP content of the fermentation broth.

#### 3.5.3. Total Flavonoid (TF) Quantification

The total flavonoid (TF) content was determined using the sodium nitrite–aluminum nitrate–sodium hydroxide color development method [72], with slight modifications. A standard curve for rutin (Y = 6.22X + 0.0054, R^2^ = 0.9955) was prepared to determine the TF content of the fermentation broth.

#### 3.5.4. Total Ginsenoside (TG) Quantification

The method described in Gavrila et al. [73] was used, with minor modification. After drying a 10 mL aliquot of the fermentation supernatant in a freeze-dryer, 0.5 g of the resultant lyophilized powder was weighed, and 5 mL of 80% methanol was added. The mixture was sonicated for 1.5 h in the KQ5200E ultrasonic cleaner set at a temperature of 50 °C (with sonication for 30 min at 10 min intervals, repeated three times). After the extractions, the extract solution was separated by centrifugation for 5 min at 10,000 rpm to obtain a TG solution. The TG solution was diluted 20 times for the determination of total ginsenoside content. A standard curve for ginsenoside Re was created (Y = 4.844X − 0.0013, R² = 0.9884). The TG content of the ginseng fermentation broth was determined using the vanillin perchloric acid method [74]. The TG content is expressed in terms of the mass of saponin Re per g of dry matter.

### 3.6. Ginsenoside Monomer Quantification

The monomeric components of the 11 ginsenosides were dissolved and diluted with 80% methanol to 55 μg/mL. They were then combined in equal proportions to produce a mixed standard solution (5 μg/mL). The TG solution was diluted 100 times, passed through a 0.22 μm membrane filter, and then sent to HPLC-MS analysis.

The chromatographic conditions were as follows: a Waters ACQUITY UPLC BEH Column (100 mm × 2.1 mm, 1.7 μm) was used, the thermostat temperature was 40 °C, the flow rate was 0.3 mL/min, and the injection volume was 1 μL. Water with 0.1% formic acid (solvent A) and acetonitrile (solvent B) was used for the mobile phase components. The gradient elution program was as follows: 0–1 min, 30% B; 1–4 min, 30–50% B; 4–11 min, 50–60% B; 11–14 min, 60–70% B; 14–18 min, 70–90% B; 18–20 min, 90% B; 20–20.2 min, 90–30% B; and 20.2–25 min, 30–30% B.

The liquid chromatography–mass spectrometry ion source operating parameters were as follows: Electrospray ion source (ESI) was used, the scanning mode was positive and negative ion switching, the multiple reaction monitoring mode was used for detection, the atomized gas flow was 3.0 L/min, the drying gas flow was 10.0 L/min, the heating gas flow was 10 L/min, the connection temperature was 300 °C, the desolvation line was at 250 °C, the heating block temperature was 400 °C, and the collision-induced dissociation pressure was 270 kPa.

### 3.7. Optimization of GF Production

#### 3.7.1. Single-Factor Screening of Culture Conditions

The following three factors were varied with the aim of increasing the antioxidant activity of the fermentation products: fermentation time (15, 30, 45, 60, and 75 h), fermentation temperature (26, 28, 30, 32, and 34 °C), and inoculum size (1.5, 2.0, 2.5, 3.0, and 3.5% *v*/*v*). The selection of time, temperature, and inoculum size as the main factors for process optimization was based on the method proposed by Tao et al. [75]. When one of the factors was being evaluated, the others were fixed at the following values: a fermentation time of 72 h, a fermentation temperature of 30 °C, and an inoculum size of 2.0% (*v*/*v*). The single-factor screening results were then used as a reference point in the response surface methodology.

#### 3.7.2. The Box–Behnken Design and Response Surface Methodology

The Box–Behnken design was used for the response surface methodology, which was used to determine the best fermentation conditions to optimize the DPPH, ·OH, and O_2_^·−^ scavenging rates. The fermentation time, fermentation temperature, and inoculum size were used as the factors. The factors and levels are shown in Table 3. A second-degree polynomial model was used to find the relationship of each factor to the response. The equation used was as follows:

(7) $$Y=\beta_{0}+\beta_{1}X_{1}+\beta_{2}X_{2}+\beta_{3}X_{3}+\beta_{12}X_{1}X_{2}+\beta_{13}X_{1}X_{3}+\beta_{23}X_{2}X_{3}+\beta_{11}{X_{I}}^{2}+\beta_{22}{X_{2}}^{2}+\beta_{33}{X_{3}}^{2}$$

where Y is the predicted response of free radical scavenging activity, X_i_ represents the independent coded factors, β_0_ is the intercept, β_i_, β_ii_, and β_ij_ are the linear coefficient, quadratic coefficient, and interaction coefficient of the polynomial model, respectively.

### 3.8. Cell Test

#### 3.8.1. Effects of the GS and GF on the Activity of HaCaT and BJ Cells

HaCaT and BJ cells were placed in 96-well plates at a density of 5 × 10^4^ cells/well with fresh medium. After 24 h of pre-culture, the medium was aspirated, varying concentrations (0.06, 0.25, and 1.00%) of the ginseng fermentation samples were added to each well, and the cells were then cultured for another 24 h. Non-treated cells maintained in culture medium were used as the control. Following incubation, the culture medium was decanted, and 100 μL of serum-free DMEM plus 10 μL of the Cell Counting Kit-8 (CCK-8) test solution was added to all wells. The cells were incubated for 1 h, and the absorbance at 450 nm was determined using a microplate reader. Cell viability was calculated using the following formula:

(8) $$\mathrm{Cell}\;\mathrm{viability}(\%)=A_{1}\;/\;A_{2}\;\times \;100$$

where A_1_ is the absorbance of the sample well, and A_2_ is the absorbance of the control well.

#### 3.8.2. Assessment of the Capacity of the GS and GF to Protect Cells Exposed to H_2_O_2_

To determine the baseline viability of HaCaT and BJ cells exposed to increasing concentrations of H_2_O_2_, a CCK-8 assay was performed. Cells in the logarithmic phase of growth were seeded in 96-well plates at 5000 cells/well. Once the cells adhered, the medium was aspirated and replaced with a medium containing varying concentrations of H_2_O_2_ (50, 100, 200, 400, and 800 μM). After a 24 h incubation period, the cells were washed with phosphate buffer saline (PBS) and treated with the CCK-8 solution. Following a 1 h incubation, the absorbance was measured at 450 nm using a microplate reader to determine cell viability.

To explore the reparative and protective effects of the GS and GF samples, stimulus repair and stimulus protection assays were used. In the stimulus repair experiments, cells in the logarithmic phase of growth were seeded in 96-well plates at 5000 cells/well. After cell adhesion, the medium was replaced with solutions containing various concentrations of H_2_O_2_. Following a 24 h stimulation period and washing with PBS, different concentrations of the GS and GF were added, and the cells were incubated for an additional 24 h. Subsequently, cell viability was assessed using the CCK-8 method. In the stimulus protection experiments, cells in the logarithmic phase of growth were seeded in 96-well plates at 5000 cells/well. After cell adhesion, different concentrations of the GS and GF were added, and the cells were incubated for 24 h. Then, a medium containing various concentrations of H_2_O_2_ was introduced, and after 24 h, cell viability was determined.

### 3.9. Statistical Analysis

All experiments were performed in triplicate, and the data were analyzed using GraphPad Prism 8.0 for Windows (San Diego, CA, USA); data are presented as the mean value ± standard deviation. Data were evaluated by one-way analysis of variance to detect statistical significance, followed by post hoc multiple comparisons (Dunn’s test). A *p* value < 0.05 was considered to indicate a statistically significant difference. Graphical abstracts were created using Figdraw 2.0 (https://www.figdraw.com/#/).

## 4. Conclusions

In this study, we utilized response surface methodology coupled with the Box–Behnken design to optimize the fermentation of a ginseng stem and leaf extract. The optimal conditions were determined and experimentally verified. The GF showed strong free-radical scavenging activity against DPPH, ·OH, and O_2_^·−^. At 0.06%, the GF was not cytotoxic and showed the dual effects of protection and repair on H_2_O_2_-treated HaCaT cells. The GF significantly increased the viability of H_2_O_2_-treated HaCaT cells, effectively protected HaCaT cells against oxidative stress-related damage, and repaired damaged HaCaT cells. Thus, the findings of this study indicate that solutions produced by fermenting ginseng stem and leaf extracts with LAB may have significant antioxidant activity and could potentially be used to delay skin aging. The findings also warrant the further exploration of the GF as a cosmetic ingredient with beneficial properties. However, this study still has certain limitations, including a lack of clarification on specific active ingredients and a limited exploration of the mechanistic aspects. Therefore, in subsequent research, major components of the GF fermentation broth will be separated and subjected to activity assays. Advantageous components will be selected for non-targeted and targeted metabolism studies to further elucidate the mechanisms related to antioxidant activity and cellular oxidative damage repair and prevention.

## Figures and Tables

**Table 1:** Test design of fermentation parameters and corresponding antioxidant activity results.

Run	A (h)	B (°C)	C (%)	DPPH (%)	O_2_^·−^ (%)	·OH (%)
1	30	28	2.50	82.79 ± 0.16	47.53 ± 1.63	75.62 ± 4.78
2	30	28	2.50	84.72 ± 2.23	49.39 ± 3.01	76.61 ± 3.56
3	45	28	2.00	73.07 ± 0.71	43.49 ± 2.57	70.10 ± 2.37
4	30	28	2.50	80.26 ± 1.52	46.09 ± 2.50	78.57 ± 3.33
5	30	30	2.00	80.56 ± 2.77	42.16 ± 4.33	75.01 ± 0.63
6	45	30	2.50	71.41 ± 1.42	44.28 ± 2.40	68.63 ± 2.01
7	30	28	2.50	81.48 ± 2.49	46.16 ± 2.16	81.39 ± 0.43
8	15	30	2.50	77.80 ± 4.98	41.15 ± 3.11	76.75 ± 1.61
9	15	28	3.00	83.54 ± 0.69	39.40 ± 3.79	77.22 ± 0.92
10	15	26	2.50	80.97 ± 2.71	36.16 ± 1.92	73.56 ± 0.23
11	30	26	3.00	83.03 ± 1.93	40.64 ± 2.57	72.98 ± 1.50
12	30	28	2.50	79.35 ± 0.33	46.02 ± 1.34	77.54 ± 0.59
13	45	26	2.50	69.89 ± 4.61	44.61 ± 1.04	76.08 ± 0.14
14	45	28	3.00	71.34 ± 2.69	40.68 ± 3.07	69.71 ± 1.68
15	15	28	2.00	82.39 ± 2.71	37.20 ± 2.59	72.25 ± 1.30
16	30	26	2.00	83.71 ± 2.76	40.23 ± 2.83	76.54 ± 1.50
17	30	30	3.00	80.63 ± 1.88	41.45 ± 1.77	72.55 ± 0.72

Note: A represents fermentation time, B represents fermentation temperature, and C represents inoculum size.

**Table 2:** ANOVA with O_2_^·−^ radical clearance as the response value.

Sources	Sum of Squares	df	Mean Square	*F*-Value	*p*-Value
Model	201.02	9	22.34	14.27	0.0010
A	45.84	1	45.84	29.28	0.0010
B	6.84	1	6.84	4.37	0.0749
C	0.1063	1	0.1063	0.0679	0.8020
AB	7.06	1	7.06	4.51	0.0713
AC	6.30	1	6.30	4.02	0.0849
BC	0.3115	1	0.3115	0.1990	0.6690
A²	43.37	1	43.37	27.70	0.0012
B²	21.88	1	21.88	13.97	0.0073
C²	55.76	1	55.76	35.61	0.0006
Residual	10.96	7	1.57		
Lack of Fit	2.47	3	0.8245	0.3887	0.7682
Pure Error	8.49	4	2.12		
Cor Total	211.98	16			
R^2^	0.9483				
R^2^_Adj_	0.8818				

Note: A represents fermentation time, B represents fermentation temperature, and C represents inoculum size.

**Table 3:** Factor levels of fermentation parameters.

Factor	Level		
	−1	0	1
A: time/h	15	30	45
B: temperature/°C	26	28	30
C: inoculum size/%	2.0	2.5	3.0

**Table 4:** Correlation analysis of GF fermentation broth: active ingredients and antioxidants.

	TS	TP	TF	TG	DPPH	·OH	O_2_^·−^
TS	1	0.500	0.737	0.822	0.189	−0.731	−0.952
TP		1	−0.217	0.904	−0.756	−0.225	−0.212
TF			1	0.221	0.803	−1.00 **	−0.908
TG				1	−0.404	−0.213	−0.609
DPPH					1	−0.808	−0.48
·OH						1	0.905
O_2_^·−^							1

** 0.001 < *p* < 0.01.

## Data Availability

The data presented in this study are included in the article.

## Supplementary Materials

The following supporting information can be downloaded at: https://www.mdpi.com/article/10.3390/molecules29061265/s1, Figure S1: Total ionization chromatogram (TIC) of GF fermentation broth; Figure S2: Effects of different concentrations of H_2_O_2_ on HaCaT/BJ cell activity; Table S1: Correlation analysis of GF fermentation broth of 11 ginsenosides and antioxidants.

## Abbreviations

DPPH, 2,2-diphenyl-1-picrylhydrazyl; ·OH, hydroxyl radical; O_2_^·−^, superoxide anion; TGs, total ginsenosides; TCM, traditional Chinese medicine; FBG, fermented black ginseng; LAB, lactic acid bacteria; CFDA, China Food and Drug Administration; GA, *Lactobacillus acidophilus* (GDMCC1.412); GB, *Lactiplantibacillus plantarum* 1.140 (GDMCC1.140); GC, *Lactobacillus casei* (GDMCC1.159); GD, *Lactobacillus rhamnosus* (ATCC7469); GE, *Lactiplantibacillus plantarum* 1.648 (GDMCC1.648); GS, unfermented ginseng broth; GF, optimized fermentation broth; DMEM, Dulbecco’s Modified Eagle Medium; pNPG, p-nitrophenyl-β-glucopyranoside; Na_2_CO_3_, sodium carbonate; p-NP, p-nitrophenol; TSs, total sugars; TPs, total phenols; TFs, total flavonoids; ESI, electrospray ion source; CCK-8, cell counting kit-8; PBS, phosphate buffer saline; PPT, protopanaxatriol; HaCaT, keratinocytes; BJ, fibroblasts; SOD, superoxide dismutase; GSH-Px, glutathione peroxidase; MDA, malondialdehyde.
