# Supplementary material for: Optimization of Antioxidant Activity of Compounds Generated during Ginseng Extract Fermentation Supplemented with Lactobacillus
Source: Molecules. 2024 Mar 13;29(6):1265. doi: 10.3390/molecules29061265 (PMC10975595; doi:10.3390/molecules29061265)
Supplement: Supplementary file 1 [file molecules-29-01265-s001.zip › Figure S2.pdf]

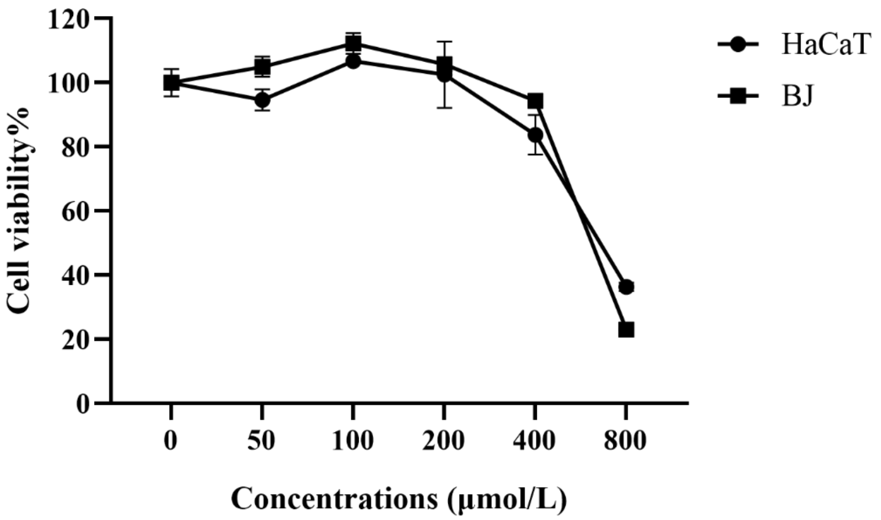

Figure S2. Effects of different concentrations of H_2O_2 on HaCaT/BJ cell activity.

Data are expressed as mean ± SD, n = 3. Values with no letters in common are significantly different ($p < 0.05$).
