# Supplementary figures and images for: Optimization of Antioxidant Activity of Compounds Generated during Ginseng Extract Fermentation Supplemented with Lactobacillus
Source: Molecules. 2024 Mar 13;29(6):1265. doi: 10.3390/molecules29061265 (PMC10975595; doi:10.3390/molecules29061265)

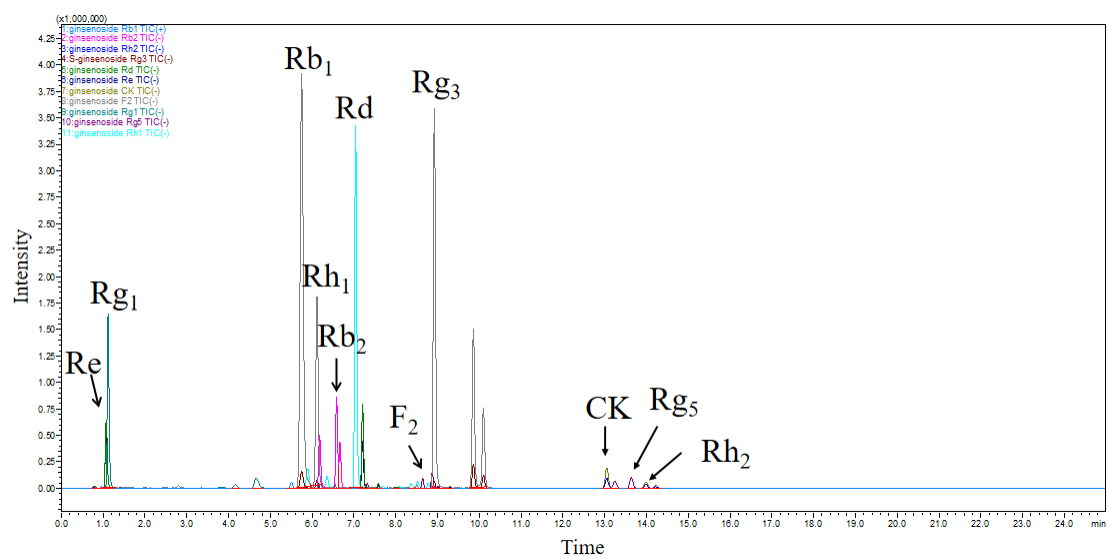

Figure S1. Total ionization chromatogram (TIC) of GF

Supplement: Supplementary file 1 [file molecules-29-01265-s001.zip › Figure S1.pdf]
